# Keratinocyte growth factor in acute lung injury to reduce pulmonary dysfunction – a randomised placebo-controlled trial (KARE): study protocol

**DOI:** 10.1186/1745-6215-14-51

**Published:** 2013-02-18

**Authors:** Laurence JM Cross, Cecilia M O’Kane, Cliona McDowell, Jospeh J Elborn, Michael A Matthay, Daniel F McAuley

**Affiliations:** 1 Centre for Infection and Immunity, The Queen’s University of Belfast, Health Sciences Building, 97 Lisburn Road, Belfast, BT9 7BL, Northern Ireland; 2 Regional Intensive Care Unit, Royal Victoria Hospital, Belfast Health and Social Care Trust, Grosvenor Road, Belfast, BT12 6BA, Northern Ireland; 3 Clinical Research Support Centre, Royal Victoria Hospital, Belfast Health and Social Care Trust, Grosvenor Road, Belfast, BT12 6BA, Northern Ireland; 4 Cardiovascular Research Institute, University of California, San Francisco, 505 Parnassus Avenue, M-917, San Francisco, CA, 94143-0624, USA; 5 Departments of Medicine and Anesthesia, University of California, San Francisco, 505 Parnassus Avenue, San Francisco, CA 94143, USA

**Keywords:** Keratinocyte growth factor, Palifermin, Acute lung injury, Adult respiratory distress syndrome, Oxygenation index, Pulmonary oedema, Respiratory failure

## Abstract

**Background:**

Acute lung injury is a common, devastating clinical syndrome associated with substantial mortality and morbidity with currently no proven therapeutic interventional strategy to improve patient outcomes. The objectives of this study are to test the potential therapeutic effects of keratinocyte growth factor for patients with acute lung injury on oxygenation and biological indicators of acute inflammation, lung epithelial and endothelial function, protease:antiprotease balance, and lung extracellular matrix degradation and turnover.

**Methods/design:**

This will be a prospective, randomised, double-blind, allocation-concealed, placebo-controlled, phase 2, multicentre trial. Randomisation will be stratified by presence of severe sepsis requiring vasopressors. Patients in an ICU fulfilling the American–European Consensus Conference Definition of acute lung injury will be randomised in a 1:1 ratio to receive an intravenous bolus of either keratinocyte growth factor (palifermin, 60 μg/kg) or placebo (0.9% sodium chloride solution) daily for a maximum of 6 days. The primary endpoint of this clinical study is to evaluate the efficacy of palifermin to improve the oxygenation index at day 7 or the last available oxygenation index prior to patient discontinuation from the study.

A formal statistical analysis plan has been constructed. Analyses will be carried out on an intention-to-treat basis. A single analysis is planned at the end of the trial. *P* = 0.05 will be considered statistically significant and all tests will be two-sided. For continuously distributed outcomes, differences between groups will be tested using independent-sample *t* tests, analysis of variance and analysis of covariance with transformation of variables to normality or nonparametric equivalents. The trial will be reported in line with the Consolidated Standards of Reporting Trials (Consort 2010 guidelines).

**Trial registration:**

http://ISRCTN95690673

## Background

Acute lung injury (ALI) is a common clinical syndrome with a reported incidence of up to 80 per 100,000 patient-years, characterised by life-threatening respiratory failure requiring mechanical ventilation and multiple organ failure, and is a major cause of morbidity and mortality [1-3]. ALI has significant resource implications, prolonging ICU stay and hospital stay, and requiring rehabilitation in the community [4-6]. The high incidence, mortality, long-term consequences and high economic costs mean that ALI is an extremely important problem.

The pathogenesis of ALI involves pulmonary neutrophil and macrophage recruitment and injury to the alveolar epithelium and endothelium, which in part determines the severity of lung injury [7,8]. The resolution of pulmonary oedema and improved outcome in ALI is associated with enhanced alveolar epithelial function, suggesting that a strategy to accelerate epithelial repair in ALI may be beneficial [9-12]. The Cochrane systematic review of pharmacological treatments that included 22 studies of 14 different drugs concluded that ‘effective pharmacotherapy for ALI is extremely limited, with insufficient evidence to support any specific intervention’ [13]. The recent publication of two studies from the National Institutes of Health National Heart, Lung and Blood Institute Acute Respiratory Distress Syndrome Network of Investigators investigating nebulised β_2_-agonist (BALTI Study) and omega-3 fatty acids with antioxidants (OMEGA study) as well as the recent publication of the UK-based BALTI-2 trial was disappointing because neither β_2_-agonists, omega-3 fatty acids, nor antioxidant supplementation was effective for ALI [14-16]. The National Heart, Lung and Blood Institute Working Group considered the future research directions in ALI in 2002 and concluded that clinical trials underpinned by mechanistic investigations were essential to develop new therapies for ALI [17].

Keratinocyte growth factor (KGF) is a 28 kDa heparin-binding member of the fibroblast growth factor family (FGF-7) and specifically binds to the KGF receptor, expressed primarily in epithelial tissues. KGF acts as a paracrine mediator of proliferation, differentiation and upregulation of cytoprotective mechanisms in epithelial cells, including alveolar type 2 pneumocytes [18]. KGF modulates several mechanisms recognised to be important in alveolar epithelial repair and therefore has become of interest as a potential therapeutic intervention in ALI. Recombinant human KGF (palifermin) is a 23 N-terminal amino acid truncated version of KGF [19]. Palifermin (60 μg/kg/day for 6 days) decreases the incidence, duration and severity of oral mucositis in patients with malignancies associated with chemotherapy and/or radiotherapy [20]. Such epithelial repair has led to interest in its potential use to treat epithelial injury in ALI [21]. There is a variety of animal models that have used KGF (between 5 and 10 mg/kg) in the setting of lung injury, in which beneficial effects were reported [22,23]. Recently, data from a human *ex vivo* lung perfusion model of endotoxin-induced lung injury indicated that treatment with KGF improved lung endothelial and epithelial barrier function and enhanced the rate of alveolar fluid clearance, hence reducing alveolar oedema [24]. The available *in vitro*, animal and human model data, as well as clinical studies in mucositis, therefore support KGF as a potential therapy for patients with ALI. We postulate that KGF may improve alveolar epithelial/endothelial barrier dysfunction, and therefore KGF may improve pulmonary dysfunction in ALI.

## Methods/design

### Trial summary

KARE is a multicentre, randomised, double-blind, allocation-concealed, placebo-controlled clinical trial. Patients fulfilling the American–European Consensus Conference Definition of ALI [25] will be randomised in a 1:1 ratio to receive an intravenous bolus of either KGF (palifermin, 60 μg/kg) or placebo (0.9% sodium chloride solution) daily for a maximum of 6 days. Randomisation will be stratified by the presence of severe sepsis requiring vasopressors.

### Outcome measures

The primary outcome is to evaluate the efficacy of palifermin to improve the oxygenation index (OI) at day 7 or the last available OI prior to patient discontinuation from the study. The OI is a physiological index for the severity of ALI, and measures both impaired oxygenation and the amount of mechanical ventilation delivered. We and others have demonstrated that the OI is independently predictive of mortality in patients with ALI [26,27]. We have chosen day 7 as we expect this time interval will minimise the competing effects of death and extubation, while allowing a sufficient time interval for biological benefit effect to occur.

The OI is calculated as follows:

(1) $$\mathrm{OI}=\left(\mathrm{mean}\;\mathrm{airway}\;\mathrm{pressure}\;\left({\mathrm{cmH}}_{2}\text{O}\right)\times {\mathrm{FiO}}_{2}\times 100\right)/{\mathrm{PaO}}_{2}\left(\mathrm{kPa}\right)$$

These simple measurements are easily and routinely collected as part of standard ventilator practice. The secondary outcomes are the OI at days 3 and 14, physiological indices of pulmonary function, as measured by respiratory compliance, and the PaO_2_/FiO_2_ ratio, at days 3, 7 and 14, as well as a change in the Sequential Organ Failure Assessment score from baseline to day 7 and 14. Safety and tolerability will be assessed by the occurrence of adverse events and suspected unexpected serious reactions. Although the duration of ventilation and ICU stay as well as ICU and hospital mortality and 28-day mortality will also be documented, these clinical outcomes are not included as major outcome measures because the study is not powered to assess these outcomes.

**Figure 1:**
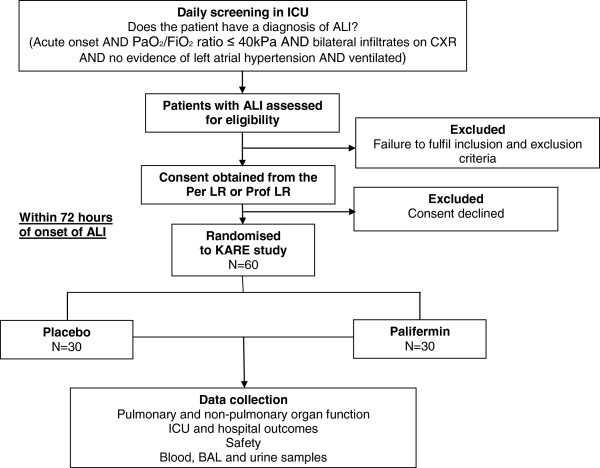
Trial schematic diagram.

The trial design is summarised in Figure 1.

### Approvals

The trial was approved by the Office for Research Ethics Committees Northern Ireland 10/NIR02/32 and the Medicines and Healthcare Products Regulatory Agency (MHRA) clinical trial authorisation number 32485/0021/001-0001 and EudraCT Number 2010-021186-70. The trial is registered on the International Standard Randomised Controlled Trial Registry (ISRCTN95690673). The trial is sponsored by the Belfast Health and Social Care Trust [28]. The trial is being coordinated by the Clinical Research Support Centre as the clinical trials unit [29]. The trial has been externally peer reviewed and is funded by the Northern Ireland Public Health Agency Research and Development Division, and will be conducted in accordance with Good Clinical Practice Guidelines, applicable UK Clinical Trials Regulations and the standard operating procedures of the sponsor. The trial will be reported in line with the Consolidated Standards of Reporting Trials (CONSORT) 2010 guidelines [30].

### Inclusion/exclusion criteria

Patients will be eligible to participate in the study if they fulfil the following inclusion criteria of ALI defined by acute onset of: hypoxic respiratory failure (PaO_2_/FiO_2_ ≤40 kPa); bilateral infiltrates on chest X-ray consistent with pulmonary oedema; no clinical evidence of left atrial hypertension or, if measured, pulmonary arterial occlusion pressure ≤18 mmHg; and requirement for positive pressure mechanical ventilation via an endotracheal tube or tracheostomy. All four of these ALI criteria must occur within the same 24-hour period. The onset of ALI is when the last ALI criterion is met. Patients must be enrolled within 72 hours of ALI onset.

Exclusion criteria included: age <18 years; >72 hours from the onset of ALI; pregnancy; participation in a clinical trial of an investigational medicinal product within 30 days; consent declined; current treatment with KGF; known hypersensitivity to palifermin or *Escherichia coli*-derived proteins; previous adverse reaction to palifermin; history of active malignancy excluding haematological malignancies; and chronic liver disease with Child–Pugh score >12.

### Trial intervention

Patients will be randomised to palifermin 60 μg/kg or normal saline placebo daily as a bolus intravenous injection for up to 6 days in 60 patients with a 1:1 randomisation. The first dose of study drug will be administered within 4 hours of randomisation and subsequent doses will be at 10:00 am daily, starting on the following calendar day.

### Study drug termination criteria

The study drug will be continued until one of the following conditions is met, whichever comes first: 6 days after randomisation (maximum treatment period); 2 days following discontinuation of assisted ventilation (unassisted breathing is defined as extubated with supplemental oxygen or room air; or open T-tube breathing; or tracheostomy mask breathing; or CPAP ≤5 cmH_2_O without pressure support – patients receiving pressure support via non-invasive ventilation will be defined as receiving assisted ventilation); study drug-related adverse event; discharge from the critical care environment; death; discontinuation of active medical treatment; patient or relative request for withdrawal of patient from the study; and decision by the attending clinician that the study drug should be discontinued on safety grounds.

### Trial procedures

#### Informed consent procedure

Informed consent will be obtained prior to conducting any trial-specific procedures.

As critically ill sedated patients do not have the capacity to give consent, consistent with requirements of the EU clinical trial directive, we will obtain written informed consent/assent from a representative in keeping with regulatory requirements before randomisation. All surviving patients will be informed about the trial at the earliest opportunity after regaining competence and consent to continue in the trial will be sought. The consent from the representative will remain valid until a decision on consent to continue is obtained from the patient.

#### Patient registration and randomisation procedure

After informed consent, the researcher will contact the clinical trials pharmacist, who will allocate a unique subject number to the patient and randomise the subject in a 1:1 ratio to the designated treatment group. The clinical trials pharmacist will dispense the trial drugs. The researcher will then contact the clinical trials unit and register the randomised patient.

The total six doses of study drug for each patient will be dispensed and stored in a secure temperature-monitored fridge. The study drug will be reconstituted and administered intravenously by appropriately trained ICU clinical staff independent of the clinical trial according to local guidelines, therefore ensuring blinding for the clinical trial staff. The ICU clinical staff who administer the study drug will not be involved in any of the study-specific assessments.

#### Standardised management

All patients will receive standardised management according to ICU guidelines with regards to feeding, antibiotic policy, fluid management and weaning. Patients will be managed using a standardised ventilation protocol aiming for tidal volumes of 6 ml/kg ideal body weight.

#### Assessments

A summary of trial procedures is shown in Table 1.

**Table 1:** Trial procedures

	**Day 1**	**Days 2 and 3**	**Day 4**	**Days 5 and 6**	**Day 7**	**Days 8 to 13**	**Day 14**
Eligibility assessment	X						
Informed consent	X						
Randomisation	X						
Baseline data	X						
Daily data		X	X	X	X	X	X
Study drug administration	X	X	X	X			
Adverse events	X	X	X	X	X	X	X
Bronchoalveolar lavage sampling	X		X				
Blood and urine sampling	X		X		X		X

#### Adverse event management

KARE is recruiting from a patient population who are already in a life-threatening situation; many of the participants will therefore be expected to experience serious adverse events. Events that are expected in this population and those that are collected as outcomes (for example, death, organ failure) of the trial will not be reported as serious adverse events. Other serious adverse events or suspected unexpected serious reactions that occur between trial entry and 30 days after the end of the trial drug administration will be reported by faxing a serious adverse event log to the sponsor.

#### End of trial

The trial will end when 60 patients have been recruited and 28-day follow-up completed.

The trial will be stopped prematurely if: mandated by the ethics committee; mandated by the MHRA; mandated by the sponsor (for example, following recommendations from the Data Monitoring and Ethics Committee); or funding for the trial ceases. Confirm and accept.

The Office for Research Ethics Committees Northern Ireland and the MHRA that issued the clinical trial authorisation will be notified in writing if the trial has been concluded or terminated early.

#### Sample size and statistical analysis

The primary outcome measure will be the difference in the OI between the palifermin-treated and placebo-treated groups at day 7 or the earliest time period prior to day 7 when the OI is available, since some patients will be extubated or will die before day 7. We have chosen day 7 because we expect this time interval will minimise the competing effects of death and extubation, while at the same time allowing a sufficient time interval for a biological effect to occur. Based on our data from a recently completed clinical trial in ALI, the mean (standard deviation) OI at day 7 in patients with ALI is 62 (51) cmH_2_O/kPa [31]. A sample size of 56 subjects (28 in each group) will have 80% power at a two-tailed significance level of 0.05 to detect a clinically significant difference of 39 cmH_2_O/kPa in OI between groups. In a previous phase 2 study of similar size, we have found that an intervention can demonstrate a change in OI of a similar magnitude [31], confirming a treatment effect of this size can be achieved.

Although we anticipate few withdrawals or loss to follow-up, we have allowed for this in the sample size calculation. In our previous single-centre study of simvastatin in ALI there were no withdrawals [31]. In a multicentre UK study of pulmonary artery catheters in ICU patients (PAC-Man), no patients were lost to follow-up, and only 2.4% withdrew consent after recovering competency [32]. A dropout rate of 5% has therefore been estimated and the study will require a total of 60 patients (30 in each group).

Using the sample size of 60 patients determined from the primary outcome measure, the differences in the secondary outcomes at day 7 [31] that can be detected between the groups are presented in Table 2. All calculations assume 80% power at a two-tailed significance level of 0.05. A statistical analysis plan has been designed and is attached as Additional file 1.

**Table 2:** Secondary outcomes

**Outcome**	**Value in patients with acute lung injury**	**Detectable effect size**
Respiratory compliance (ml/cmH_2_O)	57.8 (36.5)	27.8
Sequential organ failure assessment score	7.2 (4.2)	3.2

Data presented as mean (standard deviation).

A statistical analysis plan has been designed and is attached as Additional file 1.

## Trial status

The trial has been successfully initiated and we are recruiting patients at the Regional Intensive Care Unit Royal Victoria Hospital Belfast. There has been one major amendment to the protocol since design and ethical/MHRA approval, which has included changing from a single-site to a multisite study to ensure full recruitment to the study is achieved, a change from excluding all patients with an active history of malignancy to allow inclusion of patients with haematological malignancy, removal of the exclusion of patients with pancreatitis, extending the recruitment window to 72 hours post onset of ALI as well as the removal of pulmonary dead space measurement as a secondary objective.

## Abbreviations

ALI: Acute lung injury; FiO_2_: Fraction of inspired oxygen; KGF: Keratinocyte growth factor; MHRA: Medicines and Healthcare Products Regulatory Agency; OI: Oxygenation index; PaO_2_: Arterial partial pressure of oxygen.

## Competing interests

DFM has received consultancy fees and served on advisory boards for GlaxoSmithKline for ALI and has received lecture fees for meetings organised by AstraZeneca. The remaining authors declare that they have no competing interests.

## Authors’ contributions

DFM, CMO’K and MAM conceived the study. All authors made a substantial contribution to the protocol development. All authors have read and approved this manuscript.

## Supplementary Material

Additional file 1 Statistical analysis plan. Click here for file
