# Erratum to: Keratinocyte growth factor in acute lung injury to reduce pulmonary dysfunction – a randomised placebo-controlled trial (KARE): study protocol

**DOI:** 10.1186/s13063-017-1930-7

**Published:** 2017-05-11

**Authors:** Laurence J. M. Cross, Cecilia M. O’Kane, Cliona McDowell, Jospeh J. Elborn, Michael A. Matthay, Daniel F. McAuley

**Affiliations:** 1 Centre for Infection and Immunity, The Queen’s University of Belfast, Health Sciences, Building, 97 Lisburn Road, Belfast, BT9 7BL Northern Ireland; 2 Regional Intensive Care Unit, Royal Victoria Hospital, Belfast Health and Social Care Trust, Grosvenor Road, Belfast, BT12 6BA Northern Ireland; 3 Clinical Research Support Centre, Royal Victoria Hospital, Belfast Health and Social Care Trust, Grosvenor Road, Belfast, BT12 6BA Northern Ireland; 4 Cardiovascular Research Institute, University of California, San Francisco, 505 Parnassus Avenue, M-917, San Francisco, CA 94143-0624 USA; 5 Departments of Medicine and Anesthesia, University of California, San Francisco, 505 Parnassus Avenue, San Francisco, CA 94143 USA

## Erratum

In the original publication [1] the last sentence under the paragraph “Adverse event management” not correct. The correct version can be found here:

“SAEs that occur between trial entry and up to 28 days after completion of the study drug will be reported”.
