# Supplementary material for: Keratinocyte growth factor in acute lung injury to reduce pulmonary dysfunction – a randomised placebo-controlled trial (KARE): study protocol
Source: Trials. 2013 Feb 18;14:51. doi: 10.1186/1745-6215-14-51 (PMC3620926; doi:10.1186/1745-6215-14-51)
Supplement: Additional file 1 — Statistical analysis plan. [file 1745-6215-14-51-S1.pdf]

KARE

Keratinocyte growth factor in Acute lung injury to REduce pulmonary dysfunction – a randomised placebo controlled trial

Protocol No: 10089DMCA-CS
EudraCT Number: 2010-021186-70
ISRCTN: ISRCTN95690673

STATISTICAL ANALYSIS PLAN

Final Version 2.0

Northern Ireland Clinical Research Support Centre
1st Floor Elliot Dynes Building
The Royal Hospitals
Belfast
BT12 6BA
Tel: +44 (0)28 9063 5794
Fax: +44 (0)28 9063 3328
Email: info@crsc.n-i.nhs.uk

Version date: 15/01/2013

Contacts
Cliona McDowell – Biostatistician
Angela Toner – Data Manager

This document and all preceding versions will be stored in the Trial Master File for this trial

CONTENTS

1. Background and Design....................3

2. Outcome measures....................5

2.1 Primary outcome measure 5

2.2 Secondary outcome measures 5

3. Data....................5

3.1 CRF Forms and variables.................... 5

3.2 Management of datasets.................... 5

3.3 Data completion schedule 6

3.4 Data verification.................... 6

3.5 Data coding 6

4. Definition of terms 7

5. Sample Size Calculations....................7

6. Analysis Principles....................7

7. Analysis Details 8

7.1 Recruitment and follow-up patterns 8

7.2 Baseline Characteristics.................... 8

7.3 Trial treatment.................... 8

7.4 Trial events.................... 8

7.5 Toxicity/ Symptoms 9

8. Additional Information....................9

8.1 Trial management group (TMG).................... 9

8.2 Data Monitoring and Ethics Committee (DMEC).................... 9

9. Signatures of Approval .... Error! Bookmark not defined.

Appendix 1: summary Tables....................10

1. BACKGROUND AND DESIGN

The trial hypothesis under investigation is that treatment with palifermin will improve surrogate clinical outcomes in adult patients with ALI and is safe.

The objectives of this trial are

1) To conduct a randomised, double-blind, placebo-controlled phase 2 trial of palifermin for the treatment of ALI and
2) To study the biological mechanisms of palifermin on pulmonary and systemic neutrophil function and inflammation; alveolar epithelial and endothelial function protease:antiprotease balance and lung extracellular matrix degradation and turnover

The trial is a prospective, randomised, double-blind, placebo-controlled phase 2, clinical study of palifermin in patients with ALI.

Patients will be randomised to Palifermin 60 µg/kg or normal saline placebo daily as a bolus intravenous injection for up to 6 days. Administration will not occur through an intravenous line that has been flushed with heparin. The intravenous line will be flushed with normal saline prior to and after study drug administration. The first dose of study drug will be administered within 4 hours of randomisation and subsequent doses will be at 10 am daily starting on the following calendar day.

Patients will be eligible to participate in the study if they fulfil the following criteria:
Inclusion criteria:
ALI as defined by acute onset of:

a. hypoxic respiratory failure ($PaO2/FiO2 \leq 40$ kPa)
b. bilateral infiltrates on chest X-ray consistent with pulmonary oedema.
c. no clinical evidence of left atrial hypertension or if measured, a pulmonary arterial occlusion pressure (PAOP) less than or equal to 18 mmHg.
d. requirement for positive pressure mechanical ventilation via an endotracheal tube or tracheostomy.

All ALI criteria (a-d above) must occur within the same 24-hour period. The onset of ALI is when the last ALI criterion is met. Patients must be enrolled within 72 hours of ALI onset

Exclusion criteria:

1. Age < 18 years
2. More than 72 hours from the onset of ALI
3. Pregnancy
4. Participation in a clinical trial of an investigational medicinal product within 30 days
5. Consent declined
6. Current treatment with KGF
7. Known hypersensitivity to palifermin or Escherichia coli derived proteins
8. Previous adverse reaction to palifermin.
9. History of active malignancy excluding haematological malignancies.
10. Chronic liver disease with Child-Pugh score greater than 12.

The trial is summarised in Figure 1.

Figure 1. Trial schematic diagram

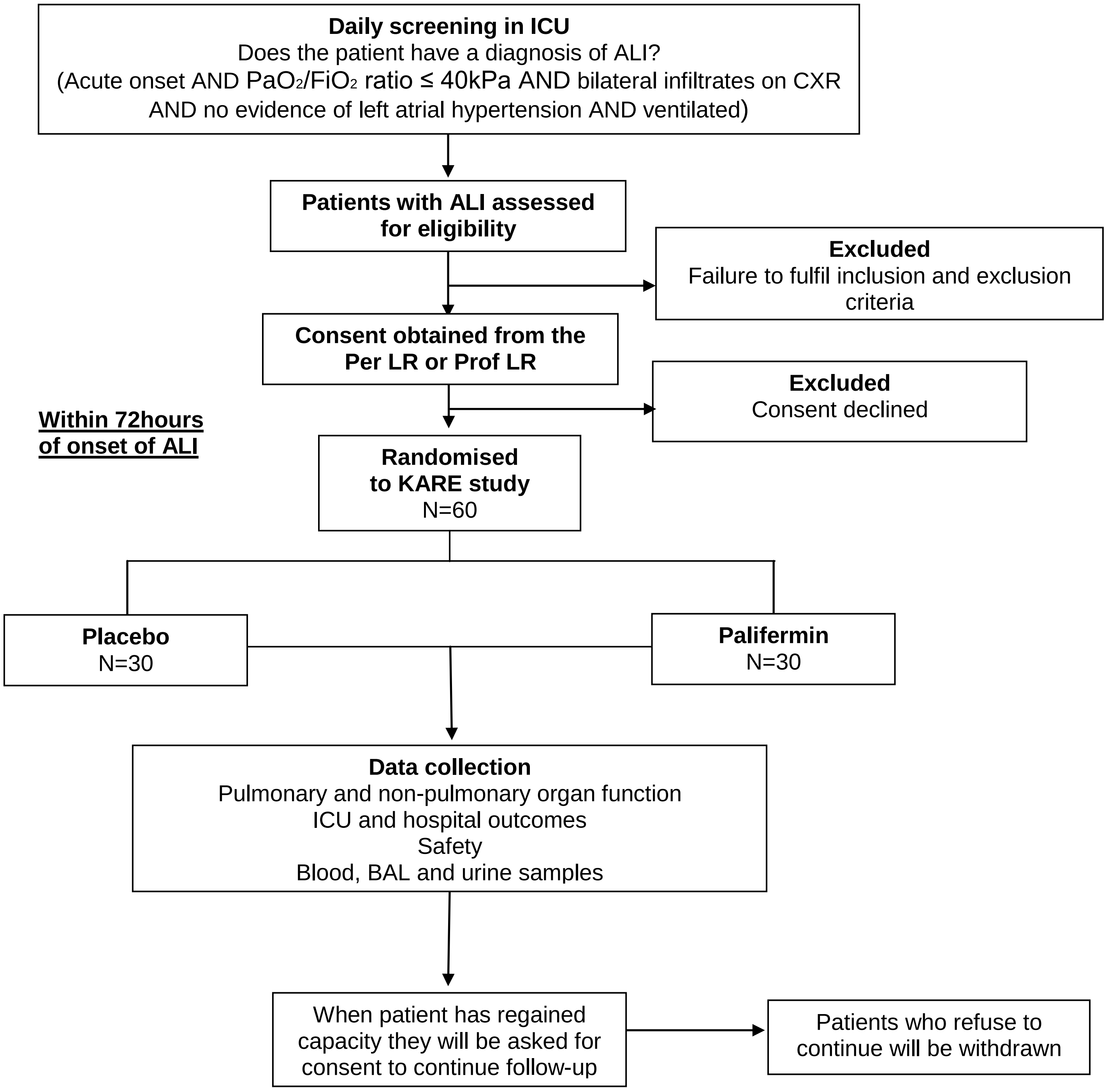

Full details of the background to the trial and its design are presented in the protocol.

2. OUTCOME MEASURES

2.1 Primary outcome measure

The primary endpoint of this clinical study is to evaluate the efficacy of palifermin to improve oxygenation index (OI) at day 7 or the last available OI prior to patient discontinuation from the study.

2.2 Secondary outcome measures

1. Oxygenation index (OI) at days 3 and 14
2. Physiological indices of acute lung injury, as measured by respiratory compliance (Crs) and P/F ratio at days 3, 7 and 14
3. Change in sequential organ failure assessment (SOFA) score from baseline to day 7 and 14
4. Safety and tolerability as assessed by the occurrence of AEs and Suspected Unexpected Serious Reactions (SUSARs).

Although the duration of ventilation and ICU stay as well as ICU and hospital mortality and 28-day mortality will also be documented, these important clinical outcomes are not included as major outcome measures as the study is not adequately powered to assess these outcomes.

The secondary objective of the study is to measure the biological effects of KGF on:

1. Systemic and pulmonary neutrophil function
2. Systemic and pulmonary inflammatory response
3. Systemic and pulmonary epithelial and endothelial function
4. Systemic and pulmonary protease and anti-protease balance
5. Pulmonary extracellular matrix (ECM) degradation and turnover

3. DATA

3.1 CRF Forms and variables

Full details of data collection and timing are described in the trial protocol (version 3.0 30 Nov 2012).
A copy of the CRF is presented in the Trial Master File.

3.2 Management of datasets

Below is the standard policy for management of data in the CRSC as given in the CTU SOPs.

At the time of analysis (including DMEC reports/Interim analysis (if required)):

The trial database will be stored in MACRO:

- In collaboration with the Statistician, the Data Manager will file out from MACRO a dataset of all data stored in the database. This will act as the frozen dataset. It is the responsibility of the statistician to accurately record the date of freezing and ensure all data is retrieved.
- New data can continue to be entered onto MACRO database.
- If any outstanding data queries are resolved during the analysis that relate to data in the frozen dataset (e.g. problems that are found during analysis or amended CRFs that

are returned to the CRSC), the main MACRO database should be changed under the oversight of the Trial Data Manager.

3.3 Data completion schedule

The following table describes the time points for completion of clinical record forms.

	Day 1	Day 2-3	Day 4	Day 5-6	Day 7	Day 8-13	Day 14
Eligibility assessment	X						
Informed consent	X						
Randomisation	X						
Baseline data	X						
Daily data		X	X	X	X	X	X
Study drug administration	X	X	X	X			
Adverse events	X	X	X	X	X	X	X
BAL sampling	X		X				
Blood and urine sampling	X		X		X		X

3.4 Data verification

Data verification, consistency and range checks will have been performed at the data entry stage by the CRSC, as well as checks for missing data (copies can be found in the Trial Master File). Additional range, consistency and missing data checks will be performed, as appropriate, when the analysis is performed (and when the datasets for analysis are constructed). All variables will be examined for unusual, outlying, unlabelled or inconsistent values.

Given the thorough nature of our follow-up procedure we expect the issue of missing data to be relatively minimal. We anticipate high compliance with initial data collection as this is close to the time of patient registration. If any data is missing imputation will not be done.

Any problems with trial data will be queried with the Trial Managers, Data Managers, or statisticians, as appropriate. If possible, data queries will be resolved, although it is accepted that due to administrative reasons and data availability a small number of problems will continue to exist. This will be minimised.

3.5 Data coding

The variable codings will be as specified on the CRF.

4. DEFINITION OF TERMS

Give definition of any terms that require explanation.

Term	Definition
Oxygenation Index (OI)	(mean airway pressure (cm H_20) x FiO_2 x 100) ÷ PaO_2 (kPa)<br>**or**<br>Mean Airway Pressure (cmH_2O) / PaO_2(kPa)/FiO_2 Ratio

5. SAMPLE SIZE CALCULATIONS

The primary outcome measure will be the difference in OI between the palifermin and placebo treated groups at day 7. Day 7 is chosen as it is expected this time interval will minimise the competing effects of death and extubation, while at the same time allowing a sufficient time interval for a biological effect to occur. Based on the data from a recently completed clinical trial in ALI, the mean (standard deviation; SD) OI at day 7 in patients with ALI is 62 (51) cmH2O/kPa. A sample size of 56 subjects (28 in each group) will have 80% power at a two-tailed significance level of 0.05 to detect a clinically significant difference of 39 cmH2O/kPa in OI between groups. In a previous phase 2 study of similar size, it was found that an intervention can demonstrate a change in OI of a similar magnitude confirming a treatment effect of this size can be achieved.
In a previous single centre study of simvastatin in ALI there were no withdrawals. In a multi-centre UK study of pulmonary artery catheters in ICU patients (PAC-Man), no patients were lost to follow up, and only 2.4% withdrew consent after recovering competency. Therefore a drop-out rate of 5% has been estimated and the study will require a total of 60 patients (30 in each group).
Using the sample size of 60 patients determined from the primary outcome measure, the differences in the secondary outcomes at day 7 that can be detected between the groups are presented in table 3. Data are mean (SD). All calculations assume 80% power at a two-tailed significance level of 0.05.

Table 3.

Outcome	Value in patients with ALI	Detectable effect size
Crs (ml/cm H20)	57.8 (36.5)	27.8
SOFA score	7.2 (4.2)	3.2

6. ANALYSIS PRINCIPLES

Standard approaches will be used to detect patterns in missing data. Analyses will be on an intention-to-treat basis. A single analysis is planned at the end of the trial. A P value of 0.05 will be considered as statistically significant and all tests will be two-sided.
For continuously distributed outcomes, differences between groups will be tested using independent samples t-tests, analysis of variance (ANOVA) and analysis of covariance (ANCOVA) with transformations of variables to Normality if appropriate, or non-parametric equivalents. Chi-squared tests (or Fisher's Exact tests) will be used for categorical variables.

Correlations between changes in the biological markers measured and physiological and clinical outcomes will assessed by appropriate graphical and statistical methods including Chi-square and Pearson's correlation coefficient.

Important time points for clinical outcomes such as OI are days 3 and 14, respiratory compliance (Crs) and P/F ratio at days 3, 7 and 14 and change from baseline in SOFA at days 7 and 14.

The randomisation is stratified by vasopressor requirement.

7. ANALYSIS DETAILS

The results of the analyses will be reported following the principle of the ICH E3 guidelines on the Structure and Content of Clinical Study Reports.

7.1 Recruitment and follow-up patterns

- Recruitment by year
- The number of withdrawals by treatment group

7.2 Baseline Characteristics

The randomisation is stratified by vasopressor requirement.
- Gender, no. (%) by treatment arm
- Vasopressor Requirement, no. (%) by treatment arm
- ALI aetiology, no. (%) by treatment arm
- Age at baseline assessment, mean (sd) by treatment arm
- Apache II score, mean (sd) by treatment arm
- SAPs II score, mean (sd) by treatment arm
- LIS, mean (sd) by treatment arm
- Listing of Baseline Medication
- Oxygenation Index, mean (sd) by treatment arm
- Respiratory Compliance (Crs), mean (sd) by treatment arm
- PaO_2/FiO_2 ratio, mean (sd) by treatment arm
- SOFA score, mean (sd) by treatment arm

7.3 Trial treatment

- Number of treatments administered
- Treatment compliance/tolerance including reasons for early discontinuation or protocol violations, no (%) by treatment arm
- Frequency and reason for dose modification and delays by treatment arm
- Additional treatments given

7.4 Trial events

- Oxygenation Index (OI) at day 7 or the last available OI prior to patient discontinuation from the study, mean (sd) by treatment arm
- Oxygenation index (OI) at days 3 and 14, mean (sd) by treatment arm
- Respiratory compliance (Crs) at days 3, 7 and 14, mean (sd) by treatment arm
- P/F ratio at days 3, 7 and 14, mean (sd) by treatment arm

- Change in sequential organ failure assessment (SOFA) score from baseline to day 7 and 14, mean (sd) by treatment arm

7.5 Toxicity/ Symptoms

- Adverse Events (AEs), Serious adverse events (SAEs) and occurrence of suspected unexpected serious adverse reactions (SUSARs), no. (%) by treatment arm

8. ADDITIONAL INFORMATION

8.1 Trial management group (TMG)

The TMG will consist of the Chief Investigator, co-investigators and staff from the CTU. The Chief Investigator will have overall responsibility for the conduct of the study. Dr Mark Cross will be responsible on a day-to-day basis for the trial. Regular meetings of the TMG will be held to discuss and solve problems and monitor progress. The Chairman of CritPaL (Barry Williams) will advise the TMG and will represent the patient's perspective ensuring that the trial remains considerate of the needs of the patients and their families.
The Chief Investigator will take responsibility for the need to change the protocol for any reason, reviewing relevant information from other sources and considering recommendations from the DMEC.

8.2 Data Monitoring and Ethics Committee (DMEC)

A DMEC will be appointed. The committee will be independent of the study team and will comprise an intensive care clinician and a clinician with experience in undertaking clinical trial. The DMEC will meet to agree conduct and remit. The DMEC will meet after the first 5 patients have been enrolled into the study and meet every 6 months thereafter. In the event of an occurrence of an unexpected severe adverse reaction an additional unplanned DMEC meeting will be convened. As this is a phase 2 trial, an interim analysis of efficacy is not planned although this issue can be discussed by the DMEC as required. The DMEC will function primarily as a check for safety, reviewing adverse events. They will report any issues pertaining to safety to the Chief Investigator. It will be the responsibility of the Chief Investigator to inform the sponsor who will take appropriate action to halt the trial if concerns exist about patient safety.

Include in the appendix any additional information that is relevant to the analyses such as:

- Additional sample size issues
- Changes in trial objectives e.g. change in comparisons groups
- Amendments to the SAP
- Example Draft Summary Tables

APPENDIX 1: SUMMARY TABLES

Table 1. Baseline Characteristics at trial entry

	**KGF**<br>**n = (%)**	**Placebo**<br>**n = (%)**
Age (years)		
Gender Male<br>Female		
Height		
Weight		
Sepsis<br>Non Sepsis		
Vasopressor Required<br>Vasopressor Not Required		
Plateau Pressure (cmH_2O)		
APACHE II score (predicted mortality %)		
SAPs score (predicted mortality %)		
LIS score		
PaO_2:FiO_2 ratio:		
Tidal Volume (ml/kg Ideal Bodyweight)		
PEEP		
Compliance		
Aetiology of ARDS<br>Direct<br>Smoke/toxin inhalation<br>Gastric content aspiration<br>Near drowning<br>Thoracic trauma<br>Pneumonia<br>Other<br>Indirect<br>Sepsis<br>Cardiopulmonary bypass<br>Pancreatitis<br>Non-thoracic trauma<br>Other		
SOFA Score		
Oxygenation Index		
Lowest Mean Arterial Pressure (mmHg)		

Mean (SD) presented for continuous variables and no. (%) for all categorical variables.

Table 2. Treatment after Trial Entry

	KGF<br>n = (%)	Placebo<br>n = (%)
Study drug given		
Time from randomisation to start of study drug (hours)<br>Mean (sd)<br>Median (IQR)<br>Range		
Reasons for termination of study drug<br>6 days after randomisation<br>2 days following discontinuation of assisted ventilation<br>Study drug related AE<br>Discharge from Critical Care<br>Request from PerLR/ProfLR/patient<br>Discontinuation of active treatment<br>Decision by a Physician on safety grounds<br>Death<br>Other		
Protocol violations:<br>Post-randomisation withdrawal<br>Refused use of data already collected<br>Refused data collection from NHS records<br>Withdrew from follow-up<br>Ineligible patient<br>Did not receive allocated treatment<br>Received treatment of other group		

Table 3: Main Clinical Outcome variables

	KGF<br>n = (%)	Placebo<br>n = (%)	Difference<br>(95% CI)	p-value
Primary outcome; change in OI at day 7 or at the last available OI prior to discontinuation*				
Change in Oxygenation Index from baseline<br>Day 3<br>Day 14				
Change in respiratory compliance<br>Day 3<br>Day 7<br>Day 14				
Change in P/F ratio<br>Day 3<br>Day 7<br>Day 14				
Change in SOFA from baseline<br>Day 7<br>Day 14				

Mean (SD) presented for treatment arms
*Median (IQR) for treatment arms
#No. (%) for treatment arms and Risk Ratio and 95% CI presented

Table 4: Safety Outcomes

	KGF	placebo	p value
Adverse events (yes/no)			
Serious adverse events (yes/no)			
SUSARs (yes/no)			

Table 5: Serious adverse events

		KGF n = (%)	Placebo n = (%)
Serious Adverse Events	Death		
	Life threatening		
	Prolongation of hospitalisation		
	Persistent or significant disability/incapacity		
	Medically important		
	Others		
Reasonable possibility caused by KARE study drug	Yes		
	No		

Table 6: Haemodynamic variables, Ventilator variables and Clinical parameters

	BASELINE KGF Pl	DAY 3 KGf Pl	DAY 7 KGF Pl	DAY 14 KGF Pl
Mean arterial Pressure				
Daily fluid balance (L)				
Frusemide (mg in 24 hours)				
PEEP				
Plateau pressure (cm H_2O)				
Mean airway pressure (cm H_2O)				
Peak pressure (cm H_2O)				
Highest Tidal volume				
PF ratio (no unit)				
OI (no unit)				
LIS (no unit)				

Table 7: Survival data and length of ventilation and stay data

	KGF	placebo	p value
14 day survival (yes/no)			
Ventilator free days (days)			
ITU free days (days)			
Duration ventilation (days)			
ITU survival (yes/no)			
Length of ICU stay (days)			
Hospital survival (yes/no)			
Hospital length of stay (days)			
All cause mortality 28 days post randomisation			
Death before discharge from ICU			
Death before discharge from hospital			

Table 8: Sepsis

	KGF	placebo	p value
Ventilator associated pneumonia (yes/no)			
Catheter related blood stream infection (yes/no)			
Nosocomial infection (yes/no)			
